# Reflex Cough

**Published:** 1881-02

**Authors:** 


### REFLEX COUGH.

Dr. Katzenbach reported a case of harassing cough in a man, aged twenty-six, of somewhat intemperate habits. He had taken cold a few days before, and had some fever, with a mucous and bloody expectoration. There was redness of the vocal cords, but no physical signs in the chest. One evening, while coughing violently, a hernia occurred. The paroxysms of coughing were most obstinate and violent. The patient grew worse for two or three weeks and lost weight, the oxalate of cerium, anodynes, quinine, and abstinence from alcohol having been tried without benefit. The cough was finally looked upon as reflex from the stomach. Codeine gr. ⅓, bismuth subnitrate gr. xv, and oxalate of cerium gr. ijss. gave immediate relief. There were no symptoms pointing to the stomach, except loss of appetite and redness of the tongue.—*New York Med. Jour.*

